# Experiences and socio-environmental contexts in the lead-up to psychosis: a qualitative analysis of the narratives of persons with psychosis from different ethnic, racial and immigrant backgrounds

**DOI:** 10.3389/fpsyt.2025.1602468

**Published:** 2025-10-07

**Authors:** Salomé M. Xavier, Adrianne Both, Els van der Ven, Manuela Ferrari, Amal Abdel-Baki, Nicole van den Bogerd, Imke Lemmers-Jansen, Srividya N. Iyer

**Affiliations:** ^1^ Prevention and Early Intervention Program for Psychosis (PEPP-Montréal), Douglas Mental Health University Institute, Montréal, QC, Canada; ^2^ Department of Psychiatry, McGill University, Montreal, QC, Canada; ^3^ Vrije Universiteit Amsterdam, Faculty of Behavioral and Movement Sciences, Clinical Developmental Psychology, Amsterdam, Netherlands; ^4^ Centre de recherche du Centre Hospitalier de l’Université de Montréal, Montréal, QC, Canada; ^5^ Clinique JAP (Jeunes adultes psychotiques), Département de Psychiatrie, Centre Hospitalier de l’Université de Montréal, Montréal, QC, Canada; ^6^ Département de psychiatrie et d’addictologie, Faculté de médecine, Université de Montréal, Montréal, QC, Canada

**Keywords:** psychosis, structural disadvantage, social determinants, immigrants, ethnic minorities

## Abstract

**Introduction:**

Previous research, predominantly quantitative, has attributed the excess risk for psychosis among immigrants and ethnic minorities to social adversity, discrimination and structural inequities. Although calls have acknowledged their potential for yielding nuanced insights, qualitative methods focused on first-person narratives have not been used in research into the social determinants of the development of psychosis.

**Methods:**

We explored the experiences and socio-environmental contexts of individuals with psychosis from diverse ethno-racial and immigrant backgrounds. We also gathered their perspectives on the causality of psychosis. We conducted in-depth interviews with 24 participants at early intervention services for psychosis in Montreal, Canada.

**Results:**

Through thematic analysis, we identified five themes: “Spaces and societies of oppression”; “Nothing to hold on to”; “Mistreated, invisible or seen in the wrong light”; “Places of freedom, connection and safety”; and “Healing and well-being”. Spaces described as oppressive fomented experiences of precarity, isolation and mistreatment. Spaces of freedom, connection and inclusivity enabled healing and well-being. Experiences of precarity, mistreatment and exclusion were more frequent for minoritized individuals. Participants attributed psychosis to multiple factors, many pertaining to social contexts.

**Discussion:**

Our findings shed light on the processes through which social contexts shape the lives and illness development of individuals from diverse backgrounds. By framing them within particular life stories and places, we gain a fuller, more fine-grained understanding of the social-structural determinants that have been identified in quantitative studies. Our work highlights the need to attend closely to patients’ social contexts and narratives and advocate for inclusivity, equity, and connection at the societal level.

## 1 Introduction

It has long been reported that some immigrant and ethnic minority populations face higher risk for psychosis than majority groups (1). This finding has been replicated in different parts of the world, with risk persisting for further generations, and being higher for racialized minorities and immigrants moving from the Global South to the Global North. There is however considerable variation in risk depending on the ethnic/immigrant group and country of residence (2–4). A critical factor for the higher risk of psychotic disorders in immigrant and ethnic minority populations is greater exposure to social adversities that are known social determinants of mental disorders (5) and of psychotic disorders (6). Exposures contributing to higher psychosis risk include determinants at the micro (individual, familial), meso (neighborhoods, communities), and macro (countries, societies) levels. At the individual level, some adverse experiences including family separation, social disadvantage (e.g., housing, financial, educational and employment instability), discrimination, marginalization, and social exclusion have been found to partially explain the higher risk of psychosis among immigrants and ethnic minorities (7, 8). Beyond individual-level social determinants, macro- and meso-level social factors pertaining to large geographical areas and neighborhoods are known to impact on psychosis risk and may also help explain risk differences between population groups. Growing up in areas of high social deprivation is associated with higher psychosis risk for the general population (9). Immigrants and ethnic minorities are more likely to live in deprived and secluded areas (10), and social deprivation may have a more pronounced impact among these populations (11). Conversely, studies on ethnic density have shown that living in areas where one’s own ethnic group comprises a high percentage of the population, along with a positive group identification, may protect from psychosis risk among ethnic minority populations, arguably by mitigating the effects of discrimination, exclusion and isolation (12). The ways in which aspects of structural disadvantage intersect and disproportionately impact ethno-racially minoritized population groups reflect issues of systemic inequity in terms of the distribution of resources, rights and opportunities and exposure to social stressors (10).

Although previous research has privileged the examination of social determinants of psychosis via quantitative approaches, in-depth qualitative studies have the potential to unravel the intricate social mechanisms involved in the development of psychosis. Being ideal for the exploration of complex social dynamics and contexts, qualitative studies allow for the generation of innovative, rich and nuanced understandings about a given phenomenon (13). Qualitative studies have already brought invaluable insights to the field of psychosis research regarding the exploration of prodromal manifestations of psychosis, the description of pathways to care and the investigation of aspects contributing to recovery (14, 15). However, very few of these studies have considered the experiences of psychosis of individuals belonging to immigrant and ethnic minority communities. When they did, important experiential differences came up, in comparison to the majority population (16–20). For instance, a study exploring pathways to care for African-, Caribbean- and European- Canadians (16) reported that feelings of guilt for not meeting family expectations, and fear of community rejection were only described by participants of African and Caribbean origin. These aspects in turn delayed care seeking. Another Canadian study reported that religious practices were described as a facilitator for recovery among Caribbean-Canadian but not European-Canadian participants (20). In a British study involving White-British and Black-Caribbean service users with psychosis, experiences of disempowerment and mistrust were particularly salient to Black-Caribbeans’ journeys through mental healthcare services (18). Aspects of stigma, lifelong social disadvantage and the absence of community support were brought up in a British study exploring perspectives on the causality of psychosis among Black African and Black Caribbean individuals with psychosis (19).

To our knowledge, no previous studies have qualitatively investigated the life experiences of individuals with psychosis from diverse ethnic, immigrant and racial backgrounds, with a focus on social contexts and, more specifically, social determinants of psychosis. This could provide a more nuanced understanding about everyday social experiences prior to the manifestation of the illness and the ways in which social contexts might have shaped the lives, well-being and development of the illness of these individuals.

## 2 Aims

This study aimed to explore the narratives, experiences, socio-cultural contexts and perspectives on the causality of psychosis of service users from diverse ethnic, racial and immigrant backgrounds in urban early intervention services for psychosis in Montreal, Canada. Our research questions were: 1) How do service users from different ethno-racial and immigrant backgrounds describe their lives before they were diagnosed with psychosis; 2) How do their experiences prior to diagnosis intersect with aspects of their social contexts; and 3) What do participants believe caused their psychosis?

## 3 Materials and methods

### 3.1 Paradigm

This study was designed from a contextualist paradigm which, by “sitting in between the two poles of essentialism and constructivism” (21, p.6), assumes an ontological stance of critical realism (22), and an epistemological position of subjectivism (23). It assumes the existence of a reality, which however may only be partially grasped, while acknowledging that meaning is co-constructed by the interpreter and the phenomenon under observation, both of which are fundamentally shaped by their social, cultural, historical and political contexts. In other words, we acknowledged that interviewed individuals’ life accounts are approximations of their everyday realities, whose interpretation and meaning is highly dependent on contexts and is co-constructed during the interview and analysis.

### 3.2 Setting and participants

Because we aimed to retrospectively assess the experiences and overarching socio-environmental contexts before the illness, we included individuals with a recent diagnosis of psychosis who were likelier to have had clearer memories of their pre-illness experiences. We recruited individuals from four early interventions services for psychosis in Montréal, Canada. These publicly funded programs have an open referral system and provide specialized assessment and treatment services for individuals with recent-onset psychosis. Together, these programs cover a vast area of Montréal, a bilingual metropolis of approximately four million inhabitants in Québec, the only French speaking province of mostly anglophone Canada. Montréal is home to a large percentage of immigrants (about 41%), both first- (25.6%) and second- generation (16.2%). Most Montrealers are francophone (63%); 11% are anglophone and 22.5% allophone (those whose first language is neither English nor French) (24)^1^.

In all programs, a multidisciplinary team provides patients a well-defined recovery-oriented treatment package for up to five years, that includes assertive case management, evidence-based psychosocial interventions and low-dose antipsychotic medication. Following internationally accepted early psychosis guidelines, these programs enroll 18-35-year-olds (excepting one program that also accepts 14-18-year-olds) experiencing a first episode of psychosis^1^ (defined as no or previous treatment with antipsychotics of no more than a specified duration of one month for three of the programs and one year for one program); and who have no diagnosis of an organic brain disorder. The present study’s inclusion and exclusion criteria were the same as those of the programs we recruited from. An additional criterion was that participants should consider themselves able to communicate in French or English.

To grasp a diversity of experiences of individuals followed in Montreal early psychosis services, a purposive sampling strategy was used. We aimed for a broad representation of genders, immigrant status, ethnic backgrounds and mother tongues (anglophones, francophones and allophones). We did not target a specific number of participants. However, with purposive sampling and our greater aim of conducting an in-depth analysis of first-person experiences in mind, we expected to interview at least 20 individuals (25). Recruitment ended when the emerging themes were considered by authors as expressing a wide range of experiences that fit the study aims, thus providing a rich and nuanced basis for in-depth analysis (26). Recruitment procedures included liaising with local research and clinical teams and displaying recruitment posters in participating programs.

### 3.3 Procedure

SX conducted in-depth interviews with participants in English or French depending on their preference (27), following a pilot-tested interview topic guide developed to address the study goals and research questions. The guide comprised broad open-ended questions and prompts for clarification and elaboration. Interviews lasted approximately one hour and were conducted in person, at the clinic (n=15) or online (n=9), depending on participants’ preferences.

The interview started with a broad exploration of participants’ life narratives throughout time until they started being followed at the early psychosis service. Interviews further explored how specific aspects pertaining to social environments (social networks, institutions, physical and social spaces) impacted (positively or negatively) participants’ lives. Finally, participants were asked about their perspective on the causality of psychosis (based on their own experience) and their opinion of previous scientific findings regarding the social determinants of psychosis risk. Basic demographics (including age, gender, self-ascribed ethnicity, and place of birth) were collected from all participants.

### 3.4 Reflexivity and positionality

The authors are women living in Canada and the Netherlands with different ethnic, immigrant, and linguistic backgrounds, working as academics in different fields (public health, epidemiology, psychology, psychiatry) and with expertise in qualitative and quantitative methodologies. The interviewer and first author of this paper (SX) is a Portuguese woman, a psychiatrist and an international doctoral student in Montréal. Some of the authors are/were clinicians (AAB and SNI) and researchers (SI, AAB and MF) based at the study sites. Throughout the study design, implementation and analysis, we strived to apply a reflexive approach that considered the different identities of all researchers involved and how these influenced the study at different junctures. While the team’s diversity allowed for different perspectives regarding the interpretations of participants’ accounts, we acknowledge that the researchers (and particularly SX, as the interviewer) may have been seen by participants as closer to the treatment team and (for those who were ethno-racially minoritized) to the White majority population. Although this may have impacted participants’ sharing, SX’s proximity in age to the participants, and her status in Canada as an international allophone student may have helped dissipate these differences/distance.

### 3.5 Analysis

Interviews were transcribed and subjected to a hybrid thematic analysis (28) using mostly an inductive approach (21). Two researchers (SX and AB) read and discussed the interview transcripts and field notes and generated initial inductive codes that were merged with super-ordinate deductive codes (generated based on the study goals and interview guide questions). An initial list of codes was grouped into categories, themes and sub-themes that comprised a preliminary codebook. The codebook was tested against all transcripts and further refined through several iterations of readings and coding. Field notes taken by SX were consulted throughout the analysis. Final themes and sub-themes were analyzed for meaning, nuances, divergences and convergences across individual participants’ narratives and between demographic groups.

Several strategies were used to enhance the study’s rigor (29), including triangulation between different data sources (interview transcripts, field notes), two different coders (SX and AB), regular debriefing between coders during the project’s implementation and data analysis, frequent consultations with project supervisors (SNI, MF, EV, ILJ, NB) and member-checking activities (scientific meetings and meetings with clinicians and service users to share preliminary results).

### 3.6 Ethics

Before any research was conducted, approval was obtained from the local Ethics Committee, and informed consent was obtained from all participants. An honorarium worth 40 CAD to compensate for time spent was provided to all participants.

## 4 Results

Twenty-four participants were recruited. 12 participants were men, 11 self-identified as an ethnic minority, 18 belonged to a linguistic minority (not francophone), and 15 were first-generation immigrants (five had refugee status or were asylum seekers). Among first- and second-generation immigrants, most were of Sub-Saharan African (n=5) and Caribbean heritage (n=5) (**Table 1**).

**Table 1:** Participants’ demographics.

Demograhic variables	N
Gender	
Man	12
Woman	11
Non-binary	1
Age group	
18-26	13
27-35	11
Immigrant status	
Yes	15
No	9
Region origin	
USA	2
Europe	3
Caribbean	5
South Asia	1
North Africa	1
Sub-Saharan Africa	5
Central and South America	1
Canada	6
Refugee status/Asylum seeker	
Yes	5
No	19
Ethnic minority status	
Yes	11
No	13
Language	
Anglophone	6
Francophone	14
Allophone*	4
Occupation	
Unemployed	6
Student	6
Employed or Job training	12
Socio-economic situation	
Comfortable	7
Meet needs with little left	13
Meet basic needs	4
Educational level	
University education	8
College-level education	5
High School	6
Less than high school	5

* Those whose first language is neither French nor English.

**Figure 1:**
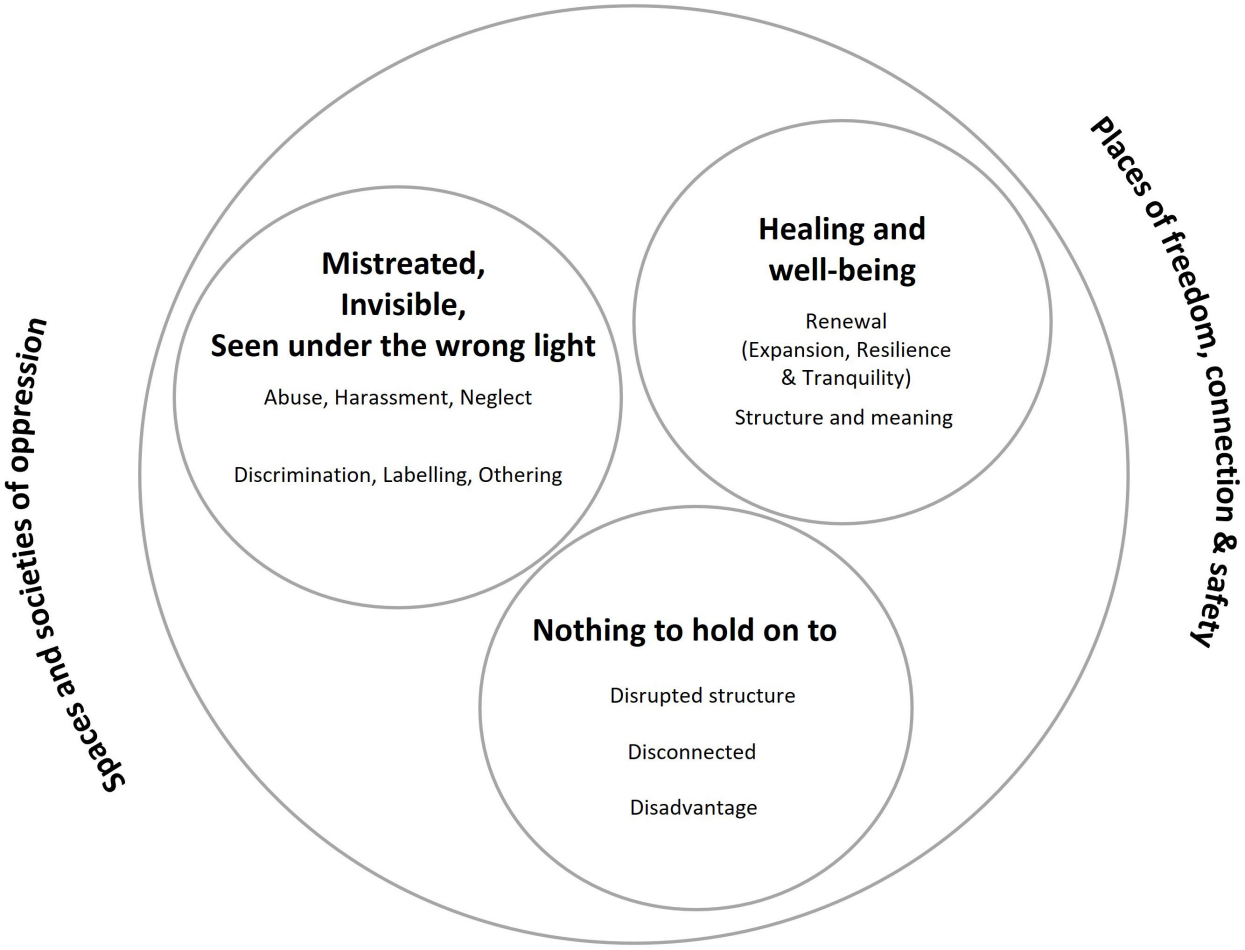
Themes and sub-themes (thematic analysis).

We identified five intertwined themes, that reflected the self-described social circumstances and experiences of participants, at different moments of their life course. The themes were: 1) Spaces and societies of oppression; 2) Nothing to hold on to (precarity, instability and isolation); 3) Mistreated, invisible or seen in the wrong light; 4) Places of freedom, connection and safety; and 5) Healing and well-being (**Figure 1**). During the interviews, participants made links between their life experiences and circumstances and the causality of psychosis. Of note, most participants described experiences or shared perspectives that could be included under most (and in many cases nearly all) the themes and sub-themes. In the results section, we will describe each theme and sub-theme, along with illustrative quotations. Basic demographic information (gender, self-ascribed ethnicity, country of birth, immigrant status, language group, in that order) is provided for each participant [P] along with the quotations. Some participants had lived in different places (besides Montréal) throughout their lives, which is mentioned when relevant.

### 4.1 Theme 1: Spaces and societies of oppression

Some participants described having lived in spaces that were experienced as suffocating, crowded, small or lacking personal space, particularly within the city. P13 (woman, Black, Caribbean, first-generation immigrant, anglophone) describes how changing living spaces from the Caribbean to Montréal as a teenager impacted her freedom and well-being, partly because of the physical constraints of her new home,

*Being in an urban environment, especially in an environment where there are winters being claustrophobic and being stuck in the house and having nothing to do, other than just, like, watching TV, it really did affect my mental health a lot. And I felt that sadness growing up. I felt that loss when I came to an urban area, and just looking at all the concrete. It was like a miracle when* sp*ring came every time, you know?*

This notion of being “stuck” was also conveyed by participants’ references to limited mobility or limited access to different areas of the city, which for P3 (man, Black, Canada, second-generation immigrant, anglophone) was a consequence of living in a deprived area of Montréal,

*Well, it’s like, um, it’s kind of like the ghetto of Montréal. So, it’s like, it’s just low-income housing and all of that stuff. So, you didn’t have [means to] commute (…) If you wanted to go to a pool, you couldn’t go. (…) It was just a low-income area. And because of that there was just like crime, you’d see glass on the streets, broken bottles, construction all the time.*

Other participants too referred to such aspects of unsafety as limiting their mobility and freedom in the city. P5 (woman, Black, Canada, second-generation immigrant, francophone) describes how living in a secluded and deprived area made her feel unsafe,

*We moved to the north part of the city. It was an apartment block, and it wasn’t very good either. There I felt even more … Less safe. There weren’t many shops in the area. We stayed in the block, so I’d say it was isolated. (…) Never seen but heard [about violence], heard stories from my neighbors. They would tell me to be careful.*

*(Translated from French)*

Some participants described spaces and structures that they had come in contact with as oppressive, due to the interplay of dynamics of inequity and exclusion. Below, P11 (woman, Black, Canada, 2^nd^ generation immigrant, francophone) describes feeling excluded after moving with her family from Montréal to a suburban area,

*So, in [Montréal neighborhood], there was a community, an immigrant community, and I had a lot of friends and everything. Then when I moved, like, I started from scratch, and there weren’t many immigrants in the suburbs. They’re more native Québecers, so it’s harder to adapt. (…) It’s very traumatic for a child to try to be included in a school where there’s just her and her sister as immigrants, and then everything else is, like, a single community that’s really closed up.*

*(Translated from French)*

Issues of inequity and injustice were mostly mentioned indirectly by participants, referring to having fewer resources, opportunities and rights than others, experiences that were often linked to being from a minoritized group and/or to a lower socio-economic stratum. In this quotation, P7 (man, Black, USA, 1^st^ generation immigrant, anglophone) explains how the dynamics of inequity in his neighborhood in the USA (where he also often experienced racial harassment) impacted his mental health growing up,

*Knowing, seeing things about other people, having, seeing how other people are having fun. It affects it, it affects the brain. And it really, I can relate to that because I didn’t have all new things coming out. New shoes, new clothes, and seeing people with those things just makes you want it. And when you want something and you can’t get it is like, it’s sad. It creates a sad feeling inside.*

Some participants also mentioned experiences of repressiveness, as they described the impact of societal limitations/restrictions on their lives. This was particularly blatant for first-generation immigrants (especially asylum seekers) or those who immigrated at a young age. They reported feeling powerless and wary about their future because of limitations associated with their (or their parents’) status and lack of work or education opportunities. Beyond issues of societal limitations or restrictions, many participants shared that they felt pressured to behave in specific ways, or to shape themselves and their lives according to societal norms. Among those born in or who grew up in Montréal, descriptions of feeling pressured to overachieve, overwork and be independent from others (including family and friends) were ubiquitous. Participants from minoritized communities described feeling these pressures more intensely because they felt they had to make extra efforts to overcome social disadvantage and structural racism. As mentioned by P3 (man, Black, Canada, second-generation immigrant, anglophone), *“It’s like you have to do double to prove yourself or something.”.*

### 4.2 Theme 2: Nothing to hold on to (precarity, isolation and instability)

Experiences of instability, precarity and isolation, mentioned by many study participants as part of their lives, were described as being enabled by oppressive contexts. Altogether, these experiences contributed to a feeling of having nothing or little to hold on to, particularly in times of need. Even if not exclusive to minoritized participants, these experiences accumulated and seemed more pervasive (more frequent and happening at different life stages) for them.

#### 4.2.1 Disadvantage (precarity)

Many described experiences of school/educational challenges, and work, financial and housing instability. In the case of P3 (man, Black, Canada, second-generation immigrant, anglophone), the struggle to find stability cut across different stages of his life, through contacts with several institutions,

*Well, after that, it’s just a mix of me going to like school, dropping outta school, going to start trying to work, getting fired, um, things like that. (…) Uh, well I dropped outta school ‘cause I, it was just, I didn’t see the point of going into it anymore. (…) I remember meeting up with this guidance counsellor and she pretty much told me, you’re never gonna be able to get into university. (…) Usually, like with most jobs, I get fired or laid off. Rarely do I get to leave.*

Experiences of homelessness, living in shelters and of contact with prison and judicial systems were mostly described by participants in the context of concomitant drug or alcohol abuse, often at a time when symptoms of psychosis had already started manifesting. For these participants, experiences of displacement, trauma (e.g., abandonment, abuse), racial and social discrimination, financial and housing instability had been present before. This was the case for P7 (man, Black, USA, 1^st^ generation immigrant, anglophone), who described at different moments of his interview how childhood experiences of racial discrimination and harassment, social inequity, and his mother’s deportation from the USA to the Caribbean (and the consequent move of the remaining family to Canada) affected his adult life, linking these experiences to his mental health struggles,

*We were discriminated because we’re African. That’s the first thing that pops into their head. And then they just make jokes about it. And then getting into school, living with my appearance. (…) And that kept, that made me depressed. (…) I was just upset most of the time. And then that upsetness, bringing that upsetness with me here in Canada, it caused me to fall into depression.*

#### 4.2.2 Disrupted structure (instability)

Several participants mentioned moments in time when sudden changes took place in their lives. Though not necessarily associated with a negative impact, when things took an unexpected turn and when control was felt as beyond one’s power, transitions were experienced as contributing to instability. With respect to changes in environment (e.g., moving across or between countries), the experience of unfamiliarity and lack of references were mentioned as destabilizing, particularly in moments of distress, and in the absence of other sources of connection and meaning. As P15 (Woman, White, Canada, francophone) describes her move from another Canadian province,

*To have changed places, to have changed jobs, to have changed institutions, to have changed my friends’ circle. It was really, like, a big change all of a sudden. And I think there’s quite a few things that have changed and clicked in my brain and made things different from what they used to be … You can’t really hold on to things as they were before, as you got used to them. So, you have to find new points of reference and quickly get attached to things.*

*(Translated from French)*

Another aspect related to the disruption of structures that came across in several interviews was the experience of interpersonal conflict, separation or loss of relevant social connections. For P16 (man, South Asian, Sub-Saharan Africa, 1^st^ generation immigrant, francophone), the separation from his fellow students at school was identified as a moment when his foundations were compromised, causing him to feel progressively isolated,

*When I was bullied at school, when I moved up to the next class, they told me that they were going to change people around. So, I wasn’t with the same gang at all and that meant that I found myself socially isolated, actually, at that time, which is to say that if I had stayed with them, maybe it could have changed things.*

*(Translated from French)*

Some participants related a general feeling of instability with personal traits of sensitivity and vulnerability, when asked specifically about their perspectives on the causality of psychosis. When they did, these issues were conflated with other biomedical explanations for psychosis (genetics, “chemical imbalances”, cannabis consumption, etc.). Importantly, for those focusing on individual-level explanations as the sole contributor to psychosis (of which cannabis consumption was a paramount example), feelings of blame, guilt, and shame also came across. However, when these aspects arose, they were usually mentioned among a myriad of other factors which could have contributed to psychosis, including socio-environmental issues,

*Well, I think the stress from relocation is a big part, stress from the war that had started, and stress from not wise kind of smoking, so all together. And I think, overall, I’m easy to disturb, I am sensitive. (…) Well, I had depressive episodes, and I had a grandmother’s sister who was diagnosed with [psychotic disorder]. So, there is, we can say, some genetic vulnerability maybe.*

(P10, woman, White, Eastern Europe, first-generation immigrant, allophone)

#### 4.2.3 Disconnected (isolation)

Several participants across demographic groups mentioned the feeling of being isolated or disconnected. For some, this was connected to the experience of feeling different from others, not fitting in or not belonging to a given group, community or environment,

*Well, when I was younger, I was often like … I don’t know how to say this but, you know, ignored by others, like, I was often in my bubble, as I said, I saw myself as apart from the world, like I don’t know why, but I had the impression that I wasn’t like other people.*

*(Translated from French)*

(P14, man, White, Canada, francophone)

Few participants linked disconnection with personality traits (a tendency to isolate or to keep to oneself), or to having been through experiences that were hard to explain to/share with others. More commonly, isolation was associated with experiences of neglect, exclusion, and marginalization. Once more, albeit not exclusive to minoritized groups, isolation in these contexts was more frequent for them.

### 4.3 Theme 3: Mistreated, invisible or seen in the wrong light

Described experiences of mistreatment ranged from clearcut episodes of physical or emotional violence/abuse (experienced or witnessed) and harassment and neglect/rejection, to everyday hostile, negative or derogatory interactions and an overall feeling of being seen by others in negative light. These experiences were often connected to experiences of precarity, isolation and instability, and described as being enabled by socio-environmental circumstances of oppressiveness.

#### 4.3.1 Abuse, harassment, neglect

Across all demographic groups, the experience of being bullied was one of the paramount examples of harassment. In the case of P13 (woman, Black, Caribbean, first-generation immigrant, anglophone), her description of bullying at school was also linked to racial and linguistic discrimination,

*I was behind in school a lot and I was bullied quite a lot because I was, I had a really thick like, English accent when I* sp*oke in French. And the teacher would always force me to read aloud in class because I guess he wanted me to improve, but it would just be like picking on me and then the kids would make fun of me. (…) And I would be walking down the stairs, and they would trip me, and they would like touch my hair when I was walking in the hallway and say like, oh you look like Minnie mouse. (…) They would ask me to do their homework or copy from the homework that I did, and I would say no a lot of the time, but then they would do things like leave trash near my locker and stuff.*

#### 4.3.2 Discrimination, labelling and othering

Several participants from minoritized groups described a range of often intersecting experiences of discrimination (racial, linguistic, gender, sexual). These were often mentioned as being at the root of experiences of harassment, exclusion and precariousness and as a source of accumulating stress and great emotional burden. In the following quotation, P3 (man, Black, Canada, second-generation immigrant, anglophone) highlights how racial discrimination impacted his everyday life,

*Like, I mean, I’m constantly feeling like I’m ridiculed, outside. I feel like when I go outside rather than be a human being, it’s like I’m this monster of a person or something. So, I think it has a lot to do with race, sometimes, but it’s something that you’re just very used to after a while. So, you kind of build up a bit of a shield towards it. So, I think after a certain amount of time it builds up and you just can’t take it anymore.*

Experiences of discrimination were not always described as blatant, but as something happening in the background of everyday life, impacting different life spheres such as work, education, romantic relationships, contacts with services and everyday interactions with friends and strangers. P5 (woman, Black, Canada, second-generation immigrant, francophone) notes how she always wondered whether racial discrimination affected her romantic relationships,

*Over the years when I couldn’t find stability, maybe that happened because I was denied things because of my color, I don’t know … It wouldn’t be blatant. But maybe it would have been easier if I had been white, maybe. (…) For example, I’ve always struggled to have good romantic relationships. And then some people told me it was … Like, my friends, they didn’t say it in a mean way, but they told me maybe it was because of my skin color. (…) That I was rejected because of that.*

*(Translated from French)*

### 4.4 Theme 4: Places of freedom, connection and safety

At different junctures during the interviews, participants mentioned the benefits of living in communities where people nurtured positive connections as a group and in places offering opportunities to engage in leisure activities and spend time outdoors and in contact with nature. Such spaces were described as being conducive to freedom, healing, well-being and safety. Similarly, social spaces that fostered inclusivity and were welcoming or accepting towards difference, were described positively,

*Because I went to [High School in Ontario], I had friends of different races, and I really enjoyed that. (…) It was a very relaxed environment, and I also felt a bit safer, hanging out with people who are also of different cultural backgrounds, just ‘cause it was easier to g*et al*ong with people.*

(P4, man, South Asian, Canada, second-generation immigrant, anglophone).

Those belonging to minoritized communities experienced being surrounded by a community where they felt represented in their identity while growing up as positive,

*As I said it can be a shock to arrive here and find everything so different, but I was lucky anyway. Sure, I wasn’t among my direct community, but the Haitian community is similar in some ways to the African community in general, and I think it helped me a lot to feel represented.*

*(Translated from French)*

(P18, man, Black, Sub-Saharan Africa, first-generation immigrant, francophone)

However, some participants pointed out that feeling part of a community entails great nuance, and sharing some commonalities (physical traits, ethnicity, culture, religion, country of origin, etc.) with others may not be enough to feel represented. Moreover, as P3 (man, Black, Canada, second-generation immigrant, anglophone) explained, individuals may still wish to integrate in other communities in their environment, while finding it hard to fit anywhere at all,

*Yeah, ‘cause it’s like you have to join [Black communities] and then you have to, not just, not that the joining part is hard, but it’s, like, integrating is really hard and just because you’re part of the same ethnic group doesn’t necessarily mean you’re going to g*et al*ong. And the same applies to like anywhere else. Let’s say I wanted to go and become something else. It’s weird because you’re kind of like the only minority there sometimes.*

For some who immigrated under adverse circumstances (e.g., refugees), keeping in contact with others from their home country was at times avoided, due to a will to start anew or as an attempt to deflect reminders of a difficult past.

### 4.5 Theme 5: Healing and well-being

Across interviews, many participants referred to sources of strength, resilience, structure and meaning in life that could be mobilized at challenging times. At times, these were mentioned as circumstances that were not present throughout life but that had to be actively sought, as part of the healing process after the onset of psychosis. These experiences were facilitated by spaces and societies characterized by freedom, connection, safety and inclusivity. In general, minoritized groups found that sources of well-being and mental health were harder to access and required greater effort to sustain, because they needed to overcome multiple and accumulating structural obstacles, often in the absence of a solid and stable support network.

#### 4.5.1 Renewal (expansion, resilience and tranquility)

Many mentioned the possibility of starting anew as being connected to feelings of hope and freedom. Interestingly, for several immigrants (especially those migrating as adults), the move to Canada itself represented this possibility. This was particularly the case for those who had encountered a community in the host country where they felt they belonged, either because it resonated with a particular aspect of their identity (gender, sexual, ethnic, cultural), or because their new community was described as hyper-diverse or generally inclusive and welcoming of difference. For some, the move to Montreal was also an opportunity to escape previous social circumstances of adversity and trauma.

Renewal was linked to the possibility of pausing, having a break and just being present, and with an overall notion of expanding one’s world. Expanding horizons was often associated with establishing connections and exploring new things in life or engaging creatively with the world (e.g., through arts, sports, leisure activities, etc.). Often mentioned in relation to expansion were notions of self-expression, authenticity (being true to oneself) and self-acceptance. For some, coming to terms with the experiences of being different from others and of having gone through adversity was a crucial step towards self-acceptance and renewed strength,

*I am very happy to be Black and I do love my experience as a Black person. I do love the richness that comes from that. I love the thinking out of the box way of living. (…) We tend to put aside what is different, but I think the fact that I am different makes me who I am. Of course, we have hardships, life will always be hard. I’m a product of hardships, but I don’t think we should let that define who we are as people, as human beings in general. (…) I say as if racism was not a thing, if sexism was not a thing, if homophobia, like if all that wasn’t there, it wouldn’t have contributed to who I am or my mental state or how I came about.*

*(Translated from French)*

(P17, woman, Black, Caribbean, francophone)

#### 4.5.2 Structure and meaning

Another aspect that many mentioned as crucial for well-being and emotional stability was the possibility of accessing sources of structure and meaning. This was often described as being possible through the connection to supportive networks such as family, friends, and communities,

*I’ve been in contact with a group of cinephiles for more than 10 years now, and we’re still a group, even if we’re fewer now, but we’ve been around for a long time, and we know each other well. (…) I’d say it’s mostly their absence that was a problem. So, the online group disappeared in 20XX and it only reappeared in 20XX. (…) And I felt like this void inside, because I did not have this connection anymore. And to have them back in 20XX, which happened after my first psychosis, was very beneficial for me.*

*(Translated from French)*

(P16, man, South Asian, Sub-Saharan Africa, first-generation immigrant, francophone)

When asked about possible aspects that could prevent psychosis or mitigate its negative consequences, participants mentioned community interventions, including mental health awareness, easier access to services, informal community mental health support structures and creation of safe spaces to connect with others,

*I think that if I had known that there were like workshops in my neighborhood, to meet other people, to work on oneself, things like that … Because I was anxious. If I had known that I had organizations in my neighborhood that dealt with this, I think I might not have had psychosis. So, I think that to reduce it, maybe there could be more programs, more exhibitions, so that people are aware that this exists. And they are not alone. Because when you’re left on your own, that’s what can happen, you can develop psychosis.*

*(Translated from French)*

(P5, woman, Black, Canada, second-generation immigrant, francophone)

## 5 Discussion

This study’s aim was to explore the experiences and social contexts of individuals with psychosis from diverse ethnic and immigrant backgrounds, along with their perspectives on the causality of psychosis. Many aspects of participants’ lives resonated with previous literature on the social determinants of psychosis, such as experiences of trauma (abuse, neglect, bullying), family separation, displacement, social disadvantage (work, housing, educational and financial instability), racial discrimination, segregation and social exclusion (7, 8, 30, 31). But, by framing them within particular life stories and places, our work yields a fuller understanding of the processes through which social contexts and social-structural determinants shape the lives and illness development of individuals from diverse backgrounds.

Physical and societal spaces described as oppressive were seen by participants as fomenting mistreatment and marginalization, as well as precarity, instability and isolation. On the other hand, spaces that fostered freedom, inclusivity and connection were described as contributing to safety, meaning, well-being and healing. Although not exclusive to minoritized groups, experiences of precarity, mistreatment and discrimination were more frequently described by and seemed more pervasive to participants belonging to minoritized groups. They were also more likely to be subjected to oppressive societal circumstances and face greater barriers to accessing sources of well-being and structure. Disconnection, alienation and isolation, whilst reported by all demographic groups, seemed more poignant among minoritized participants. This is understandable, given that experiences of displacement, othering, and marginalization, that are all more common among minoritized communities, were described by many participants as being at the root of exclusion and isolation.

Participants’ accounts of factors that possibly contributed to or attenuated the risk of psychosis cut across all themes of our analysis. Seldom was a single aspect mentioned as causal. Rather, participants endorsed a myriad of contributing factors, many of which pertained to social contexts. When not mentioned directly in relation to psychosis, socio-environmental contexts were considered at the very least as contributing to altered emotional states or other forms of psychological suffering.

Participants’ contributions also added nuance to previous predominantly quantitative findings. For instance, experiences of discrimination were not always described as blatant but rather as existing in the background, or as micro-aggressions (32), affecting everyday life beyond work-related opportunities and occasional contacts involving strangers, such as close friendships and romantic relationships. Importantly too, belonging to ethno-racially minoritized groups was not always associated with feeling marginalized, particularly by those who had come from places where they had experienced even greater inequity and segregation or who had migrated in the wake of social adversity or trauma. As reported previously, participants referred to the benefits of growing up in a community with which they shared a common cultural heritage or ethnic background (12). However, our findings also highlighted the potential benefits of populational hyper-diversity (33), when it is a valued aspect of society. There is a need to further explore the local circumstances that enable feelings of belonging and safety within culturally diverse communities. Our results also suggest that processes of identification and acculturation will always be contingent on aspects that go beyond the individual to how others define one’s identity, how one’s own group is perceived, and whether and how a society’s policies and structures integrate diversity (34).

Living in close-knit communities or in contact with nature were mentioned as positive and conducive to wellbeing. This was associated with the propensity of these areas to increased social connection and a sense of space, safety and freedom, which may be difficult to attain in dense, crowded, impersonal and isolating spaces where privacy, tranquility, leisure and safety may be challenged (35). Importantly, the experience of living spaces depended on where in the city people lived, at which stage in their lives and in which socio-economic circumstances, and on the availability of a local social network. It also depended on the multiple intersecting aspects of identity (gender, preferred language, migration history, self-ascribed ethnicity, etc.) and how they played out in a given societal context, namely, through the institutions that participants had contact with (10). This is a reminder that, in examining the role of contexts, the physical and social aspects of space ought not to be studied in isolation from one another. Relatedly, large-scale societal issues were found to greatly impact life trajectories. Across different demographic groups, societal spaces were experienced as oppressive and burdensome when they limited and restricted freedom, fostered inequity or injustice and/or exerted pressure towards a given normativity (with a focus on individuality and productivity). These findings are aligned with previous research on the impact of inequity on mental health (36), and with research that problematizes individualism and productivity in neoliberal societies (37). Moreover, our findings resonate with previous studies that highlight the relevance of intersecting systems of unfavorable social circumstances and oppression and how they disproportionately impact on those who do not hail from dominant groups (5, 38).

### 5.1 Limitations

The choice of recruiting participants from services may have resulted in a less diverse picture with respect to both types of experiences and perspectives. This excluded those who may not have been able to access services, did not find services appropriate for themselves and/or found alternative sources of healing. Their social experiences and views of causality may have been substantially different from those of the recruited participants. For example, our recruitment strategy could have resulted in a preponderance of participants favoring biomedical and psychological models of suffering and illness. Few participants described aspects of spirituality and religiosity either as explanations for psychosis or as aspects contributing to well-being and healing, even though these have been reported in previous studies (39, 40). That our participants were served at publicly funded clinics and interviewed by a White European researcher may have made them likelier to align with Québec’s militantly secularist worldview, that banishes religion from public spaces and relegates it to the private sphere, and hesitant to discuss the role of religion in their experience of psychosis (41, 42). That we included only persons who could speak English or French may have excluded first-generation immigrants who felt closer to the culture of their country of origin, who might have had different experiences and explanatory models. Further, this study and the applied methods and tools (e.g.: interview topic guide) were built in the backdrop of Eurocentric ways of knowing and doing, which in turn are based on very specific notions of personhood, society, health and illness (43, 44). All these aspects are likely to have molded participants’ explanations about their illness to fit a Eurocentric perspective. Still, our questions were open-ended and worded without reference to any clinical or medical language, which mitigates this concern.

Although we aimed to recruit individuals from diverse backgrounds, many participants belonging to minoritized groups were of African or Caribbean origins. Interestingly, these are also the groups that have been reported to be at higher risk for psychosis and of facing harsher pathways to care in different geographic locations, including in Canada (45, 46). Nonetheless, our study included individuals who were considerably diverse in terms of gender, language, migration and ethnic minority background. This resulted in rich and diverse accounts in terms of narratives and social experiences. Whether our findings appertain particularly to the lead-up to psychosis among immigrants and ethnically diverse individuals or more generally to the very experience of immigration or ethnic minority status is unclear. The recommendations that stem from them, however, are no less relevant for this uncertainty. Another important limitation is that participants have not been involved in the reviewing or writing of this manuscript. However, the preliminary results of this study were shared with people with lived experience of psychosis, and their comments and inputs were considered in the interpretation and discussion of results.

### 5.2 Implications

Our findings highlight the potential of and need for future studies focused on different environmental exposures, informed by theories of eco-social and structural intersectionality (38). They also call for further qualitative work that explores specific findings in greater detail, e.g., how ethnic density influences risk for and experiences in psychosis.

From a clinical practice and services’ perspective, our findings underline the need for service providers to acknowledge and actively address issues of power and structural inequities, to pay closer attention to social contexts and narratives of their patients, and to establish closer connections with community bodies and resources. Great improvements could come from a commitment to a context-informed, person-centered and patient-perspective approach, based on respectful curiosity and humility, and stemming primarily from the perspective and preferences of patients (47). Broadening the focus of clinical interventions, including the intake interview, could create space for different illness explanations to emerge that account for the role played by the larger contexts of individuals’ lives. The resulting narratives may better reflect the complex interplay of individual, social, cultural and contextual factors, be less blaming of the individual (or their flawed chemistry or personality) and therefore easier for service users to integrate into their life narrative. These various steps are relevant for establishing trust in therapeutic relationships. Importantly, they can help the field shift away from a dominant culture of inequity and structural disadvantage that particularly impacts minoritized communities and seems to pervade mental healthcare services, as evinced by the harsher pathways to care, higher disengagement and worse outcomes that some minoritized populations suffer (46, 48, 49).

On a larger scale, our findings highlight the continued need to focus on social determinants of (mental)health at the macro- and meso-level, which have great potential to inform public health interventions (50). Moreover, our results should also be seen as an encouragement to dismantle walls between healthcare and community spaces and services, and to advocate for spaces that foster healing, well-being and mental health within and outside healthcare services. In general, societies should work harder at the macro- and meso-level to create better life conditions for their communities, promote inclusivity, tolerance and equity of resources and opportunities for all citizens, and create spaces and structures that counter isolation and promote socialization, creativity, dialogue and mutual support. These efforts could build on existing recommendations, that draw on knowledge of public health interventions that address the social determinants of (mental) health (5). Such changes would contribute to greater societal equity and positively impact on the well-being and mental health of everyone, not only those at risk for psychosis.

## Data Availability

The datasets presented in this article are not readily available because the original contributions presented in the study are included in the article. Further inquiries can be directed to the corresponding author and will be analyzed on a case by case basis, upon reasonable request, to preserve participants’ confidentiality. Requests to access the datasets should be directed to srividya.iyer@mcgill.ca.

## Footnotes

1 Diagnosis of a non-affective psychotic disorder (e.g., schizophrenia) or affective psychotic disorder (e.g., bipolar disorder) (as per DSM-V criteria), based on the evaluation of a trained psychiatrist.
